# BET inhibition induces synthetic lethality in PTEN deficient colorectal cancers via dual action on p21^CIP1/WAF1^

**DOI:** 10.7150/ijbs.91867

**Published:** 2024-03-11

**Authors:** Guowen Ren, Jinghong Chen, Yue Pu, Eun Ju Yang, Shishi Tao, Pui Kei Mou, Li-Jie Chen, Wenli Zhu, Kin Long Chan, Guanghui Luo, Chuxia Deng, Joong Sup Shim

**Affiliations:** 1 Cancer Centre, Faculty of Health Sciences, University of Macau, Taipa, Macau SAR, China.; 2 MOE Frontiers Science Center for Precision Oncology, University of Macau, Taipa, Macau SAR, China.; 3 Central laboratory, the Second Affiliated Hospital, Guangzhou Medical University, Guangzhou, Guangdong, China.; 4 Kiang Wu Hospital, Macau SAR, China.

**Keywords:** Colorectal cancer, PTEN, synthetic lethality, BET, MYC, p21, AKT, Phosphorylated p21 at Thr145

## Abstract

Loss of PTEN tumor suppressor is an important event during colorectal cancer (CRC) development and is a target for therapeutic exploitation. This study reports that bromodomain and extra-terminal motif (BET) is a synthetic lethal partner of PTEN in CRC. BET inhibition (BETi) selectively induced G1 cell cycle arrest and apoptosis in *PTEN^-/-^* CRC. Further, BETi selectively and dose-dependently suppressed the growth of *PTEN^-/-^* CRC tumor xenografts in mice and patient-derived organoids. Mechanistically, PTEN-deficient CRC cells elevated the level of cytoplasmic p21^CIP1/WAF1^ that is hyper-phosphorylated at Thr145 by AKT. BETi suppressed AKT activation in PTEN-deficient CRC cells, followed by the reduction in p21 phosphorylation at Thr145, thereby promoting its nuclear translocation. In addition, BETi suppressed MYC level and this in turn increased the total p21 level in the nuclei. Over-expression of a phospho-mimetic p21 mutant (T145D) significantly rescued the BETi effect on PTEN-deficient CRC. These results suggest that BETi has a dual action on p21: elevating the level of p21 by inhibiting MYC and converting the oncogenic (cytoplasmic) p21 into the tumor-suppressive (nuclear) p21 by inhibiting AKT. Taken together, this study identified the synthetic lethal interaction between PTEN and BET, and provides a potential actionable target for CRC with PTEN loss.

## Introduction

The PI3K/AKT signaling cascade is crucial to cell growth and survival, which is often constitutively activated in malignant tumors. Receptor tyrosine kinases (RTKs) recruit PI3K to the membrane and this in turn stimulates PDK1 and AKT, contributing to activation of multiple downstream effector molecules, including those regulating cell metabolism, proliferation, survival and migration 1. PTEN, as a dual phosphatase, is the primary negative regulator of PI3K/AKT signaling network by dephosphorylating phosphatidylinositol 3,4,5-trisphosphate (PIP3). *PTEN* gene is located on chromosome 10q23 where the deletion is frequently observed in most cancer 2. Hence, loss-of-function mutations of PTEN present in a wide spectrum of cancers. In colorectal cancer (CRC), PTEN loss is reported to occur in approximately 34.5% of all the cases, demonstrating its crucial role as a tumor suppressor in CRC. PTEN loss results from a mix of genetic and epigenetic mechanisms, and is associated with the disease progression, metastasis, drug resistance and poor prognosis and survival in CRC patients 3-5.

Current therapeutic approaches for targeting PTEN-deficient cancer are primarily focused on developing inhibitors of PI3K/AKT kinases. A number of specific or dual kinase inhibitors of PI3K/mTOR or AKT inhibitors have been developed but only a few of them have been introduced into the clinic in the last decade. Most of the developing inhibitors in this pathway could not reach the clinic, largely because the use of these kinase inhibitors as monotherapies ended with a poor clinical outcome. The occurrence of drug resistance to the inhibitors of PI3K/AKT kinases is another major clinical challenge because PI3K and AKT inhibition often activates parallel signals or induces feedback-mediated activation of upstream RTKs 6. Therefore, diverse strategies to directly target PTEN loss have emerged recently, including functional restoration of PTEN activity and synthetic lethality to target PTEN loss in cancer cells 7.

AKT (also known as protein kinase B) is a serine/threonine protein kinase that plays central roles in promoting cell proliferation and survival, as well as in glucose metabolism and cell migration in PTEN-deficient cancer 8. One of its downstream substrates is the cyclin-dependent kinase inhibitor 1 (CDKN1), also known as p21 (CIP1/WAF1). In nucleus, p21 functions as a tumor suppressor by strictly controlling G1-S cell cycle transition and regulating PCNA-dependent DNA replication. In contrast, cytoplasmic p21 functions as an oncogene by promoting cyclin/CDK complex and inhibiting apoptosis 9. AKT-mediated phosphorylation of p21 at Thr145 is known to play a crucial role in cytoplasmic retention of p21 and its oncogenic functions 10. Increased level of cytoplasmic p21 in AKT-activated cancer cells contributes to cancer cell survival and drug resistance, suggesting the role of oncogenic p21 in apoptosis-resistant phenotypes of PTEN-deficient tumors 11.

With an aim at discovering novel actionable targets in PTEN-deficient CRC, we performed synthetic lethality drug screens against a highly selective compound library comprising 318 highly-specific small molecule inhibitors of various human druggable targets and identified several potential synthetic lethal candidates, including MDM2 inhibitors 12. In this study, we report that bromodomain and extra-terminal motif (BET) inhibitors are synthetic lethal with PTEN deficiency in CRC cells. BET is a family of bromodomain-containing epigenetic reader proteins that bind to specific acetylated lysine residues at histone tails and regulate transcription of their target genes 13. BET family proteins emerged as therapeutic targets for cancer treatment due to their function in activating transcription of some crucial oncogenes, such as MYC 14. We further found that BET inhibition converted oncogenic p21 to tumor-suppressive p21 in PTEN-deficient CRC, leading to a selective induction of G1 arrest and apoptosis in the cells. This study provides a novel actionable target for the treatment of PTEN-deficient CRC with a plausible mechanism.

## Materials and methods

### Cell culture

HCT116 and DLD1 colorectal Cancer (CRC) cell lines were acquired from American Type Culture Collection (ATCC, U.S.A) and cultured under 37 °C with 5% CO2 in a humidified incubator. All cell lines were cultured in Gibco RPMI 1640 (Thermo Fisher Scientific, Waltham, MA) with 1% Penicillin/Streptomycin (P/S; Gibco)) and 10% fetal bovine serum (FBS; Gibco).

### Generation of *PTEN*-knockout (KO) HCT116 and DLD1 cell lines

HCT116 and DLD1 cells were transfected with PTEN CRISPR/Cas9 KO Plasmids and Homology-Directed Repair (HDR) plasmids pairs (Santa Cruz Biotechnology) using Lipofectamine 3000 (Thermo Fisher Scientific, Waltham, MA) regent. The CRISPR/Cas9 KO Plasmids contain GFP gene, and the HDR plasmids contain RFP and puromycin-resistance gene for KO clone selection. PTEN KO clones were selected with RFP signal and puromycin (1 μg/ml). The PTEN KO isogenic cells were validated with immunoblot assay.

### Cell viability analysis

The cell viability was assessed with AlamarBlue reagent (Sigma-Aldrich, St. Louis, MO). Cells were incubated with AlamarBlue solution at 10% for 3 h and measured fluorescence signal (excitation at 560 nm and emission at 590 nm) with SpectraMax-M5 (Molecular Devices, Sunnyvale, CA). OTX-015 (DC7150), IBET151 (DC5183), LY294002 (DC1058) and MK-2206 (DC7465) were all purchased from DC Chemicals (Shanghai, China). The cell images were collected by the IncuCyte ZOOM (Essen BioScience, Ann Arbor, MI).

### Highly Selective Inhibitor Library screening

Highly selective inhibitor library containing 318 small molecule inhibitors with individual specific targets was purchased from Selleck Chemical. Each inhibitor was diluted in 8 doses and loaded into 384 well-plates prior to cell seeding. HCT116 *PTEN*^+/+^ and *PTEN^-/-^* cells were seeded into two sets of drug libraries separately for each screening. Cell viability was measured with AlamarBlue reagent after 4 days of drug incubation. The screening was repeated twice. A half-maximum inhibitory concentration (IC_50_) of each inhibitor for each cell type was calculated, and the synthetic lethal candidates were selected by the ratio of IC_50_ for PTEN-proficient cells to IC_50_ for PTEN-deficient cells, expressed as Selectivity Index (SI, SI=IC_50_ *PTEN^+/+^*/IC_50_ *PTEN^-/-^*). Inhibitors with SI>2 were considered as potential synthetic lethal candidates for further verification.

### Colony formation staining

Approximately 1,000 cells/well of HCT116* PTEN* isogenic cells were plated in 6-well plates and treated with compounds or dimethyl sulfoxide (DMSO). After 10 days of incubation, cells were washed with phosphate-buffered saline (PBS) and stained with crystal violet. Colonies of each group were counted using Image J software.

### Hoechst33342 staining

Approximately 5×10^5^ cells were seeded in 6-well plates and were incubated until fully attached. Cells were treated with compounds or DMSO for 24 h. Then the cells were incubated with 1 μg/ml Hoechst33342 for 30 min in a 37 °C incubator. The cell images were taken with EVOS (Invitrogen, Thermo Fisher Scientific).

### Small interfering RNA gene silencing

The siRNA transfection was performed with reverse transfection method using Lipofectamine RNAi MAX reagent (Thermo Fisher Scientific). Briefly, siRNA at different concentrations was diluted in 10 μl Opti-MEM (Thermo Fisher Scientific, USA) and mixed with 10 μl Opti-MEM containing 0.05 μl Lipofectamine RNAi MAX reagent, and the transfection mixture was incubated at room temperature for 5 min. Cell solutions (3,000 cells per well in a 96-well plate) were added to each well containing the transfection mixture. Cells were then incubated for 72 h, and cell images were taken with IncuCyte ZOOM (Essen BioScience, Ann Arbor, MI). Cell viability was assessed with AlamarBlue assay, and the siRNA sequences are shown in Supplementary Table 1.

### Plasmids and transfection

pCDH-puro-MYC plasmid was a gift from Jialiang Wang (Addgene plasmid #46970) 15, and Flag p21 WT and T145D plasmids were gifts from Mien-Chie Hung (Addgene, plasmid # 16240 and 16242) 16. The plasmids used were transfected by the forward transfection method. Briefly, the solution A was prepared with Lipofectamine 3000 (Thermo Fisher Scientific) and Opti-MEM (Thermo Fisher Scientific, USA), and the solution B was prepared with plasmid and Opti-MEM. After 5 min mixing of A and B solution, the mixture was added dropwise into the media in each well containing cells. After 24 h, cells were treated with compounds or DMSO. The cell viability was measured with AlamarBlue assay after 72 h treatment, and the transfection efficiency was analyzed by immunoblot assay.

### Western blotting and antibodies

Whole-cell proteins were extracted with 2× Laemmli sample buffer (65.8 mM pH6.8 Tris-HCl, 26.3% (w/v) glycerol, 2.1% SDS and 0.01% bromophenol blue). Proteins were equally loaded to SDS-PAGE (polyacrylamide-electrophoresis) gels and transferred onto a polyvinylidene fluoride (PVDF) membrane for immunoblotting with a primary antibody, followed by the incubation with the HRP-conjugated secondary antibody (Santa Cruz Biotechnology) for 1 h at room temperature. The target proteins were detected with Clarity^TM^ Western ECL Substrate (Bio-Rad) under ChemiDoc^TM^ MP Imaging System (Bio-Rad). All the primary antibodies used in this study were listed in Supplementary Table 2.

### Tumor xenograft mouse model

All animal experiments were approved by the Animal Research Ethics Committee of the University of Macau and carried out in accordance with the ARRIVE (Animal Research: Reporting In Vivo Experiments) guidelines 17. HCT116 and DLD1 *PTEN^+/+^* and* PTEN^-/-^* were subcutaneously injected in the left and right flanks of female BALB/c nude mice with six-week-old age. After the tumor volume reached 50-100 mm^3^, the mice were randomly divided into three groups and treated with vehicle alone (sterile saline containing 5% DMSO, 5% Tween-80 and 5% PEG-400) or OTX015 (10 mg/kg and 20 mg/kg in vehicle) by intraperitoneal injection daily for 28 days. The tumor volume and mouse body weight were measured 3 times per week. All the tumor volumes were measured by a Vernier caliper and calculated according to modified ellipsoid formula (tumor volume = long axis × short axis^2^ × π/6). At the end of the experiment, mice were sacrificed, and the tumor tissues were collected for weighting and further analysis.

### Patient-derived organoid model

Colon cancer and paired blood samples were obtained from the Kiang Wu Hospital, Macau. Prior patient written consents were acquired from donors with informing the use for organoid culture, drug testing, genomics sequencing, publication, and other associated scientific studies. This study was assessed and approved by the ethics committees of University of Macau and Kiang Wu Hospital.

The patient-derived organoids (PDOs) were collected and cultured following previously established culture conditions 18. Briefly, the fresh tumor tissues were digested with digestion buffer at 37 °C for about 1 h with gentle shaking, and then centrifuged at 2000 rpm for 3 min, washed with DMEM supplemented with 10% FBS, and spun down again at 2000 rpm for 3 min. After treating with RBC lysis buffer (eBiosciences) for 3 min, the remaining tumor cells were collected for organoid culture and cryopreservation. Dissociated tumor cells were resuspended in Matrigel (Corning) solution, then seeded in prewarmed culture plates at 31 μL per drop. Once cell Matrigel drops were solidified at 37 °C, 2.5 ml/well organoid culture medium was added in to initiate continuous culture. Medium was refreshed every 2~3 days and passage developed every 5~10 days. For drug response assay, the organoids were supplemented with 2.5% Matrigel and seeded in type-I collagen gel precoated 384-well plate at a density of about 3,000 cells per well. The third day, drugs were added into each well at 6-point serial dilutions (0.12-300 μM) and incubated for 4 days. The organoid viability was examined with the CellTiter-LumiTM Plus assay (Beyotime). For protein expression analysis, the dissociated cells were passaged and cultured in a 6-well plate for two days before drug treatment. The total protein of organoids was extracted with RIPA buffer (10 mM Tris‐HCl pH 8, 140 mM NaCl, 1 mM EDTA, 0.5 mM EGTA, 1% Triton, and 0.1% SDS) after 24 h treatment and analyzed with previous methods.

### Flow cytometry analysis

For cell cycle analysis, cells were collected and then fixed with 70% ethanol overnight at -20 °C. Cells were then incubated with RNase A (100 μg/ml) and propidium iodide (5 μg/ml) to stain the DNA. For apoptosis analysis, cells were harvested and re-suspended in Annexin V and PI staining buffer at room temperature for 15 min according to the protocol of FITC-Annexin V Apoptosis Detection Kit with PI (BioLegend, San Diego, CA). The stained cells were analyzed with a BD Accuri C6 flow cytometer (BD Biosciences, San Jose, CA) with a standard flow cytometry protocol 19.

### Real-time quantitative PCR

Cells were lysed with a cell lysis buffer (10 mM Tris pH 7.4, 0.25% IGEPAL CA-630, and 150 mM NaCl). Cell lysates containing RNA samples were then instantly subjected to reverse transcription using High-Capacity cDNA Reverse Transcription Kit (Thermo Fisher Scientific). Taq Universal SYBR Green Supermix (Bio-Rad, USA) was used for quantitative PCR analysis. All primers used in this study were synthesized by BGI Genomics (Hong Kong) and the sequences are shown in Supplementary Table 3.

### Immunofluorescence assay

Cells were seeded into 8-well chambered slides (Thermo Fisher Scientific). After treatment, cells were fixed with 4% paraformaldehyde at 37 °C for 30 min, permeabilized with 0.2% Triton X-100 and incubated in 3% BSA blocking solution for 30 min to block non-specific bindings of antibody. Cells were then incubated with a primary antibody diluted in 3% BSA in PBS overnight at 4 °C. Then, the cells were washed with PBS for 5 min 3 times and incubated with Alexa Fluor 488- or 594-conjugated-secondary antibodies (Thermo fisher Scientific) for 1 h at room temperature in the dark. Cell nuclei were stained with 1 μg/ml Hoechst33342 for 30 min. The fluorescence images were acquired by Zeiss LSM 880 Confocal system (Carl Zeiss, Thornwood, NY).

### Statistical analysis

Statistical significance of differences between two groups was determined by Student's t test using GraphPad Prism 6 (GraphPad Software, La Jolla, CA). Error bars represent the standard deviation (SD) of a mean. All statistical tests were two-tailed and *P* values <0.05 were considered significant.

## Results

### Inhibition of BET induces synthetic lethality in PTEN-deficient colorectal cancer cells

**Figure 1:**
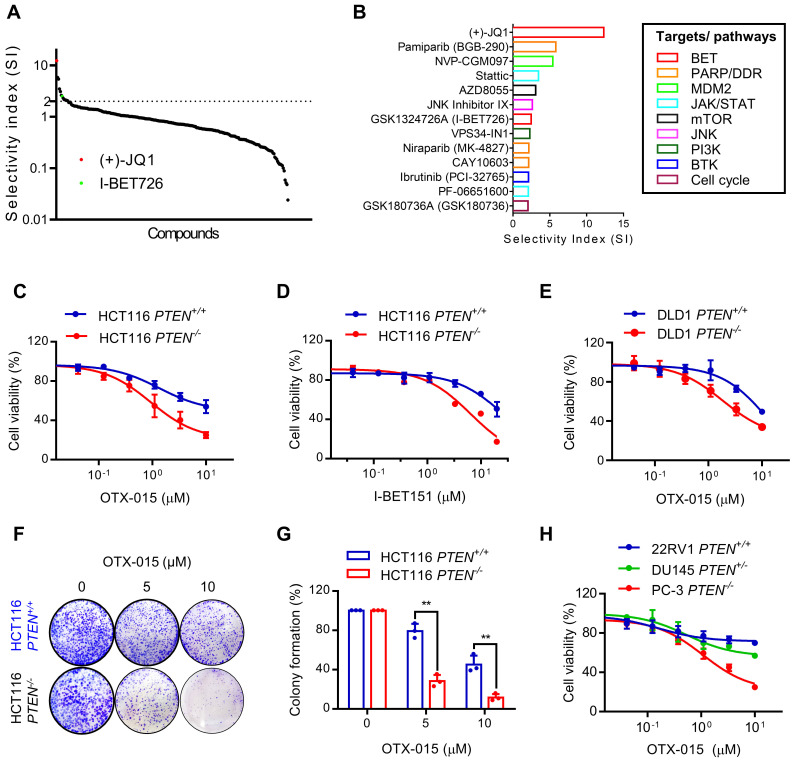
** BET inhibition induced synthetic lethality in *PTEN*-deficient CRC cells. A** Selectivity indices (SI= IC_50_ -*PTEN^+/+^*/ IC_50_ -*PTEN^-/-^*) of the drugs against HCT116 *PTEN^+/+^* and* PTEN^ -/-^* cells are shown. The identified two BET inhibitors are shown with red and green dots. **B** The identified synthetic lethal candidates with SI >2 and their targets or pathways were shown. **C-E** Dose response curves of HCT116 *PTEN* isogenic cell pair treated with OTX-015 (**C**) and I-BET151 (**D**) for 72 h. **E** Dose-response curves of DLD1 *PTEN* isogenic cell pair treated with OTX-015 for 72 h. **F** Image of colonies in six-well-plate for colony formation assay. HCT116* PTEN^+/+^* and *PTEN^ -/-^* cells were exposed to OTX-015 (0 μM, 5 μM, 10 μM) for 10 days and finally stained with crystal violet. **G** The cell colonies were counted by image J, results were representative of triplicate biological experiments. Error bars represent s.d. Data are mean ± SD of three independent experiments. **P < 0.01, student's t-test. **H** Cell survival analysis from 22RV1, DU145 and PC-3 prostate cells after treatment with OTX-015 for 72 h.

To discover PTEN synthetic lethal targets in *PTEN* specific context, we previously established PTEN-isogenic HCT116 and DLD1 CRC cell lines using CRISPR/Cas9 gene knockout and conducted synthetic lethal drug screen with the 318 highly selective small molecule inhibitors for over 100 druggable targets 12. From the analysis with selectivity index (SI), we identified total 13 candidates that presented SI above 2, including BET inhibitors (JQ1 and I-BET726), PARP or DNA damage repair (DDR) pathway inhibitor (Pamiparib and CAY106-3), MDM2 inhibitor (NVP-CGM097), JAK/STAT pathway inhibitors (Stattic and PF-06651600), mTOR inhibitor (AZD8055), PI3K inhibitor (VPS34-IN1), BTK inhibitor (Ibrutinib) and cell cycle inhibitor (GSK190736A) (Fig. 1A, B). BET inhibitors (BETi) were amongst the top hits that showed greater selectivity toward *PTEN^-/-^* HCT116 over the *PTEN*^+/+^ HCT116 cells (Fig. 1A, B). Synthetic lethal effect of BETi was further validated using other two specific BETi, OTX-015 and I-BET151. We observed that these BETi selectively inhibited the growth of *PTEN^-/-^* HCT116 and DLD1 over the *PTEN*^+/+^ counterparts (Fig. 1C-E). A long-term colony forming assay also showed a selective anti-proliferative effect of BETi on PTEN-deficient CRC cells (Fig. 1F, G). Prostate cancers are another type of cancer where a high frequency of PTEN inactivation is observed 20. We therefore tested three prostate cancer cell lines with different *PTEN* status: 22RV1 (*PTEN^+/+^*), DU145 (*PTEN^+/-^*), and PC3 (*PTEN^-/-^*) for the drug sensitivity to BETi. The result indicated that PTEN-null PC3 cells exhibited the highest sensitivity to BETi, while PTEN-wildtype 22Rv1 cells were most resistant to BETi among the three cell lines (Fig. 1H). PTEN heterozygote DU145 cells exhibited marginal sensitivity to BETi. These results demonstrate that BET inhibition is synthetic lethal with PTEN inactivation in CRC and this effect could be applicable to other types of cancer, such as prostate cancer.

### BET inhibition induces G1 cell cycle arrest and apoptosis in PTEN-deficient colorectal cancer cells

**Figure 2:**
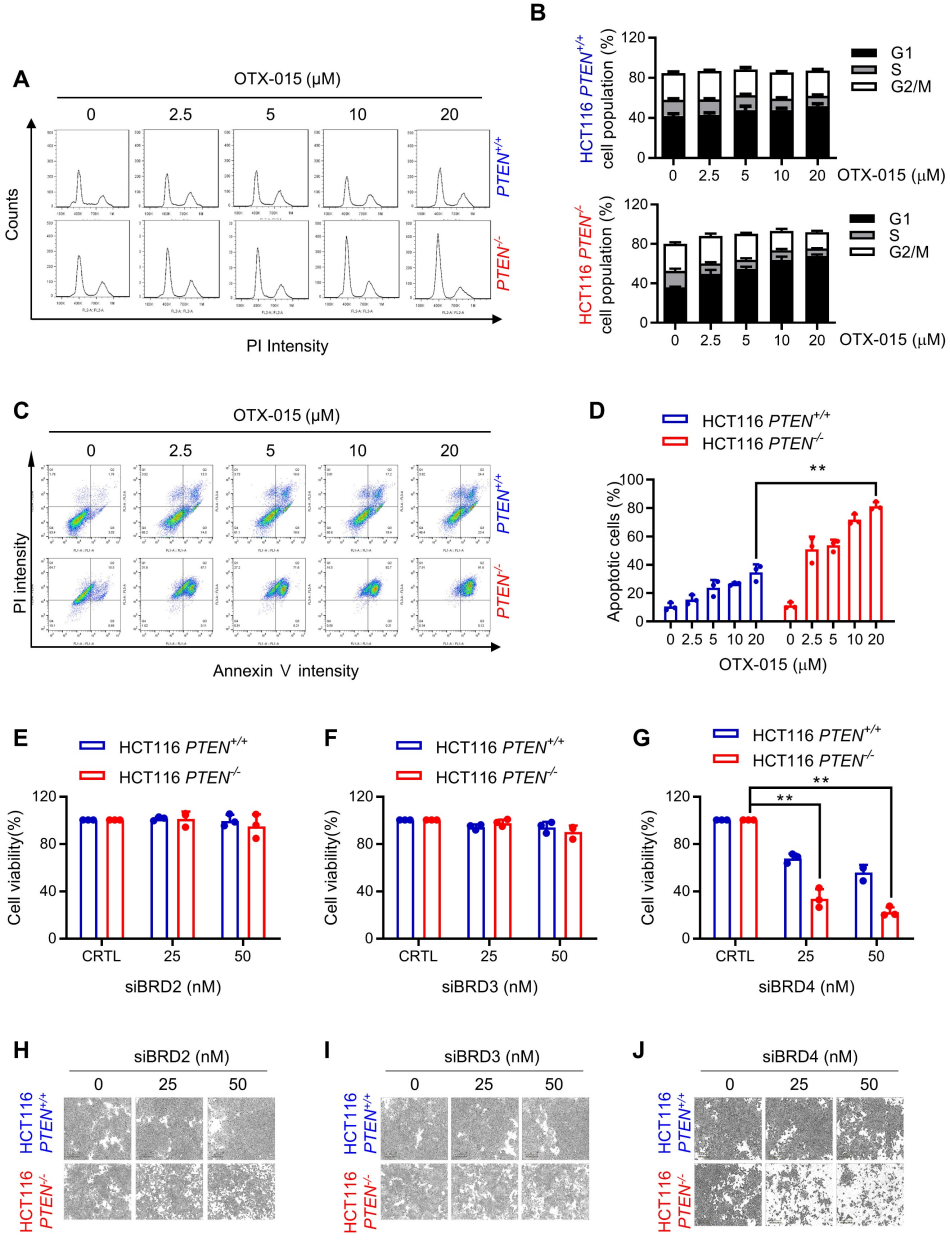
** BET inhibition induced G1 cell cycle arrest and apoptosis in *PTEN*-deficient CRC cells. A** Cell cycle analysis of HCT116 *PTEN* isogenic cell pairs after OTX-015 treatment.** B** Percentage of cell populations in each cell cycle phase. **C** Apoptosis analyses of HCT116 *PTEN* isogenic cell pairs with or without BETi treatment subjected to FITC-Annexin V and PI double staining detection with a flow cytometer. Note that the high basal PI intensity of *PTEN^ -/-^* cells is due to the RFP fluorescence signal of the *PTEN^ -/-^* cells. **D** The percentage of apoptotic cells were quantitated. **E-G** Cell survival data from HCT116 *PTEN* isogenic pair transfected with BRD2, BRD3, BRD4 siRNA. siRNA effect in each cell line was normalized to its own control cells (100%) and the bar graph was plotted independently for each cell line. **H-J** Microscope images of HCT116 *PTEN* isogenic pairs transfected with BRD2, BRD3, BRD4 siRNA. Error bars represent s.d. Data are mean ± SD of three independent experiments. **P < 0.01, student's t-test.

To investigate the mechanism of anti-proliferative effect of BETi on PTEN-deficient CRC cells, we analyzed cell cycle and apoptotic markers in cells treated with BETi. BETi dose-dependently increased the cell population at G1 cell cycle in PTEN-deficient CRC cells, but not in PTEN wildtype ones (Fig. 2A, B). Moreover, BET inhibition heavily and selectively induced apoptosis in PTEN*-*deficient CRC cells (Fig. 2C, D). Apoptotic nuclear morphology and PARP1 (Poly [ADP-ribose] polymerase 1) cleavage experiments also showed the selective apoptosis induction in PTEN*-*deficient CRC cells by BETi (Supplementary Fig. S1A, B). We then conducted a target validation experiment for BETi to rule out a possibility of off-target effects of BETi for the synthetic lethality. We used siRNAs specific for three major BET family proteins, including BRD2, 3, and 4, and analyzed synthetic lethal effects of the siRNAs on PTEN*-*deficient CRC cells. The depletion of either BRD2 or 3 did not show any meaningful effect on cell viability, but BRD4 depletion showed a synthetic lethal effect on PTEN*-*deficient CRC cells (Fig. 2E-J). These data suggest that BRD4 is the major target of BETi responsible for its synthetic lethal effect on PTEN*-*deficient CRC cells.

### MYC inhibition plays a key role in the BETi-induced synthetic lethality in PTEN-deficient colorectal cancer cells

**Figure 3:**
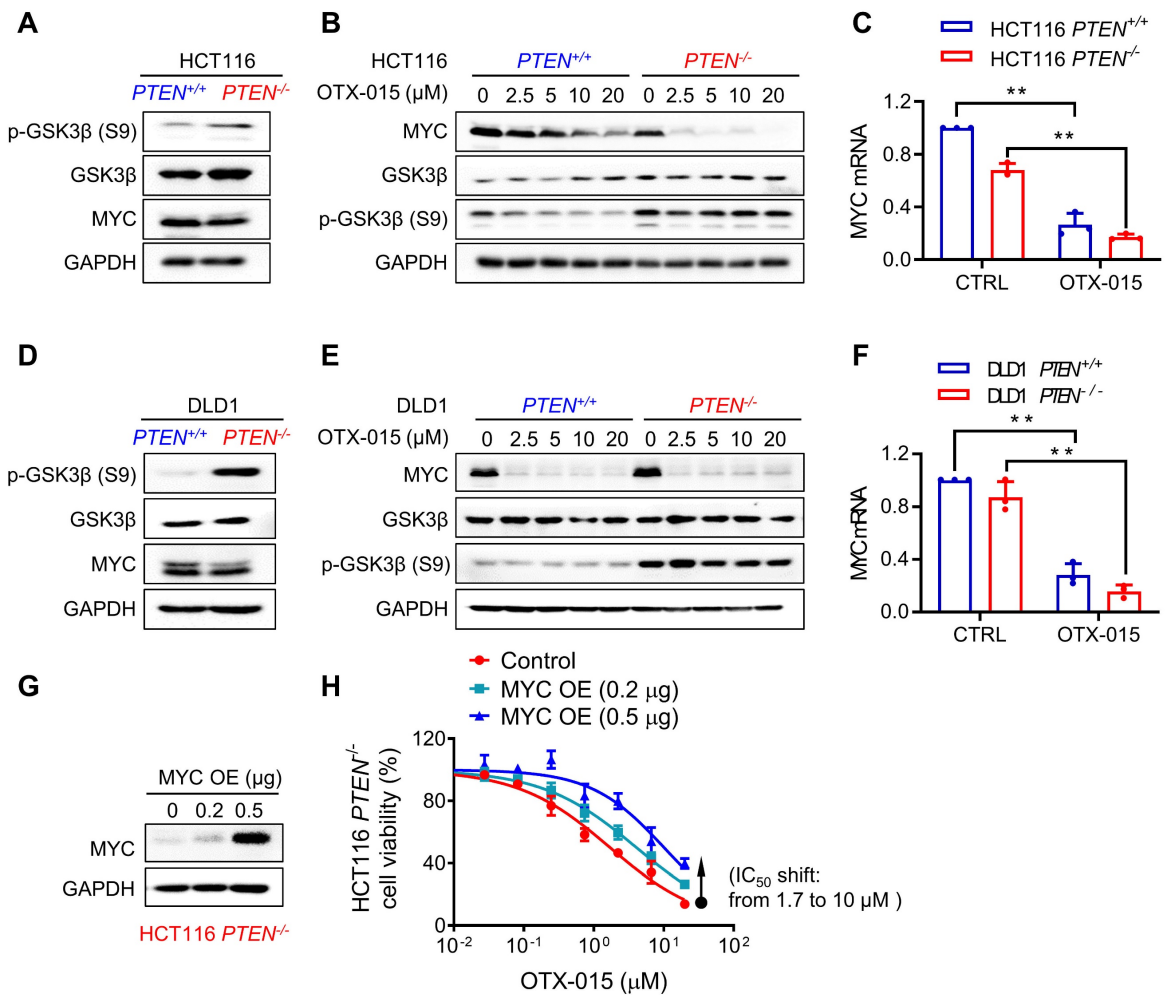
**MYC played a role in the synthetic lethality of PTEN and BET.** Western blot illustrating MYC, GSK3β and p-GSK3β(s9) levels in HCT116(**A**) and DLD1(**D**) *PTEN* isogenic cell pairs. **B, E** Western blot illustrating MYC, GSK3β and p-GSK3β(s9) levels in HCT116 (**B**) and DLD1 (**E**) *PTEN* isogenic cell pairs via treatment with OTX015 for 24 h. **C, F** RT-qPCR analysis of MYC mRNA level in HCT116 (**C**) and DLD1 (**F**)* PTEN* isogenic cell pairs. **H** Immunoblotting results reflected the MYC over-expression efficiency. **G** Survival curves of MYC over-expression *PTEN^-/-^* cell lines treated with BETi.

Based on our cell cycle and BRD4 siRNA data and previous studies describing the roles of PTEN and BET in cell cycle regulation, we hypothesized that the three proteins, including MYC, GSK3β and p21^CIP1/WAF1^ could possibly be involved in BETi-induced G1 arrest and apoptosis in PTEN-deficient CRC cells. BET family proteins are known for their roles in promoting the expression of oncogenes, with MYC being one of major transcriptional targets of BRD4 21. MYC protein stability is known to be regulated by PTEN-PI3K/AKT pathway via GSK3β 22. MYC is a key regulator of cell cycle progression by promoting the expression of cyclins and cyclin-dependent kinases (CDK) and repressing CDK inhibitors, such as p21 for G1-S transition 23. Our first hypothetic scenario was that PTEN-deficient CRC cells activate AKT, thereby inhibiting GSK3β by phosphorylation (Ser9/21) and increasing MYC protein stability 24. The elevated MYC level in PTEN-deficient CRC cells facilitates cellular MYC oncogene addiction, which can be counteracted by the MYC inhibitor BETi, thus inducing G1 arrest. We observed that GSK3β phosphorylation at Ser9 was increased in *PTEN*^-/-^ HCT116 cells, but MYC protein level was not increased (Fig. 3A, B). Cycloheximide-chasing experiment also showed that PTEN status did not affect MYC protein stability in CRC cells (Supplementary Fig. S2A). On the other hand, MYC protein level was dose-dependently decreased by BETi in both *PTEN*^+/+^ and* PTEN*^-/-^ HCT116 cells (Fig. 3B). BETi-induced MYC inhibition occurs at the transcription level as verified with MYC mRNA level and the protein stability analyses in the PTEN-isogenic cell pair treated with OTX-015 (Fig. 3C; Supplementary Fig. S2B). Very similar results were observed in the DLD1 PTEN-isogenic cell pair treated with BETi (Fig. 3D-F). To test whether MYC inhibition plays a role in BETi-induced synthetic lethality, we performed a rescue experiment by over-expressing MYC in *PTEN*^-/-^ HCT116 cells. MYC over-expression significantly reversed the anti-proliferative effect of BETi on the PTEN-deficient CRC cells, with a maximum IC50 shift from 1.7 to 10 μM (Fig. G, H). These results suggest that AKT-GSK3β-MYC oncogene addiction hypothesis is not involved in the synthetic lethality, but BETi-induced MYC inhibition still plays a key role in the synthetic lethality in PTEN-deficient CRC cells.

### PTEN inactivation increases p21 protein stability and BET inhibition increases p21 transcription

**Figure 4:**
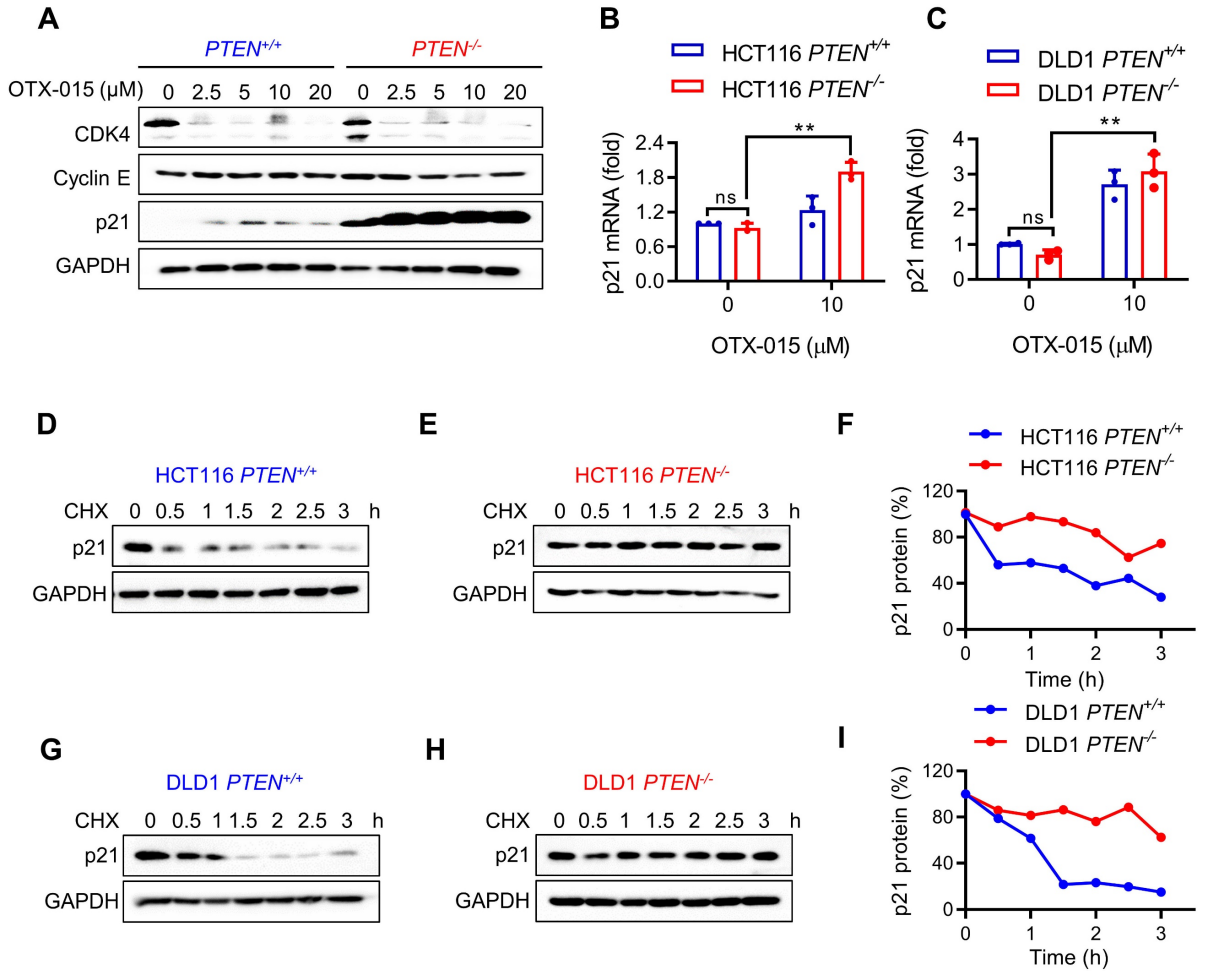
**p21 was stabilized by PTEN deficiency and further increased by BETi. A** Western blot illustrated protein levels of CDK4, Cyclin E and p21 in HCT116 *PTEN* isogenic cell pair with BETi exposure. **B, C** RT-qPCR analysis of p21 mRNA levels in HCT116 (**B**) and DLD1(**C**) *PTEN* isogenic cell pairs. Error bars represent s.d. **P < 0.01 between control and BETi treatment groups, two-way ANOVA. Cells were treated with cycloheximide (10 μM) for the times indicating, and the immunoblotting indicated p21 protein levels; **D, E** for HCT116 *PTEN* isogenic pairs, **G, H** for DLD1 *PTEN* isogenic pairs. Graph represented the data from densitometric quantification as ratio of p21/GAPDH and normalized to the "0 CHX control" for each lane; **F** for HCT116, **I** for DLD1.

Although MYC inhibition by BETi is essential for the synthetic lethality, MYC inhibition occurred in both *PTEN*^+/+^ and* PTEN*^-/-^ CRC cells in a similar extent, suggesting that there could be another factor down-stream of MYC that causes hypersensitivity toward *PTEN*^-/-^ CRC cells. Thus, we analyzed the levels of MYC target genes that are relevant to cell cycle regulation, such as CDK4, cyclin E and p21. CDK4 and several cyclins are MYC targets for transcription activation, while p21 is a MYC target for transcription repression 25. Among the MYC targets tested, the p21 level exhibited the greatest difference between *PTEN*^+/+^ and* PTEN*^-/-^ CRC cells in response to BETi treatment (Fig. 4A). The p21 protein level was significantly elevated in PTEN-deficient cells compared to PTEN-wildtype cells and was further increased by BETi treatment (Fig. 4A). To test whether the p21 elevation occurs at the transcription or post-translational level, we analyzed the p21 transcripts and protein half-life in PTEN-isogenic cell pair treated with or without BETi. RT-qPCR analysis of p21 mRNA showed that there was no meaningful difference in the level of p21 transcripts between *PTEN*^+/+^ and* PTEN*^-/-^ CRC cells (Fig. 4B and C). This result suggests that PTEN deficiency increases p21 expression at the post-transcriptional level. However, BETi treatment significantly increased p21 mRNA level in both PTEN-isogenic cells (Fig. 4B and C). This result implying that BET inhibition increases p21 transcription likely via BETi-induced MYC inhibition. In order to investigate how PTEN deficiency regulates p21 protein level, we analyzed the difference in p21 half-life between *PTEN*^+/+^ and* PTEN*^-/-^ CRC cells using cycloheximide (CHX)-chasing experiments. p21 half-life in PTEN-wildtype CRC cells was about 1 h, but that in PTEN-deficient CRC cells was significantly lengthened (>3 h) (Fig. 4D-I). These data suggest that PTEN-deficiency increases p21 protein stability, and BET inhibition further increases p21 level in PTEN-deficient cells via activating p21 transcription.

### PTEN inactivation increases p21 protein stability through AKT activation

**Figure 5:**
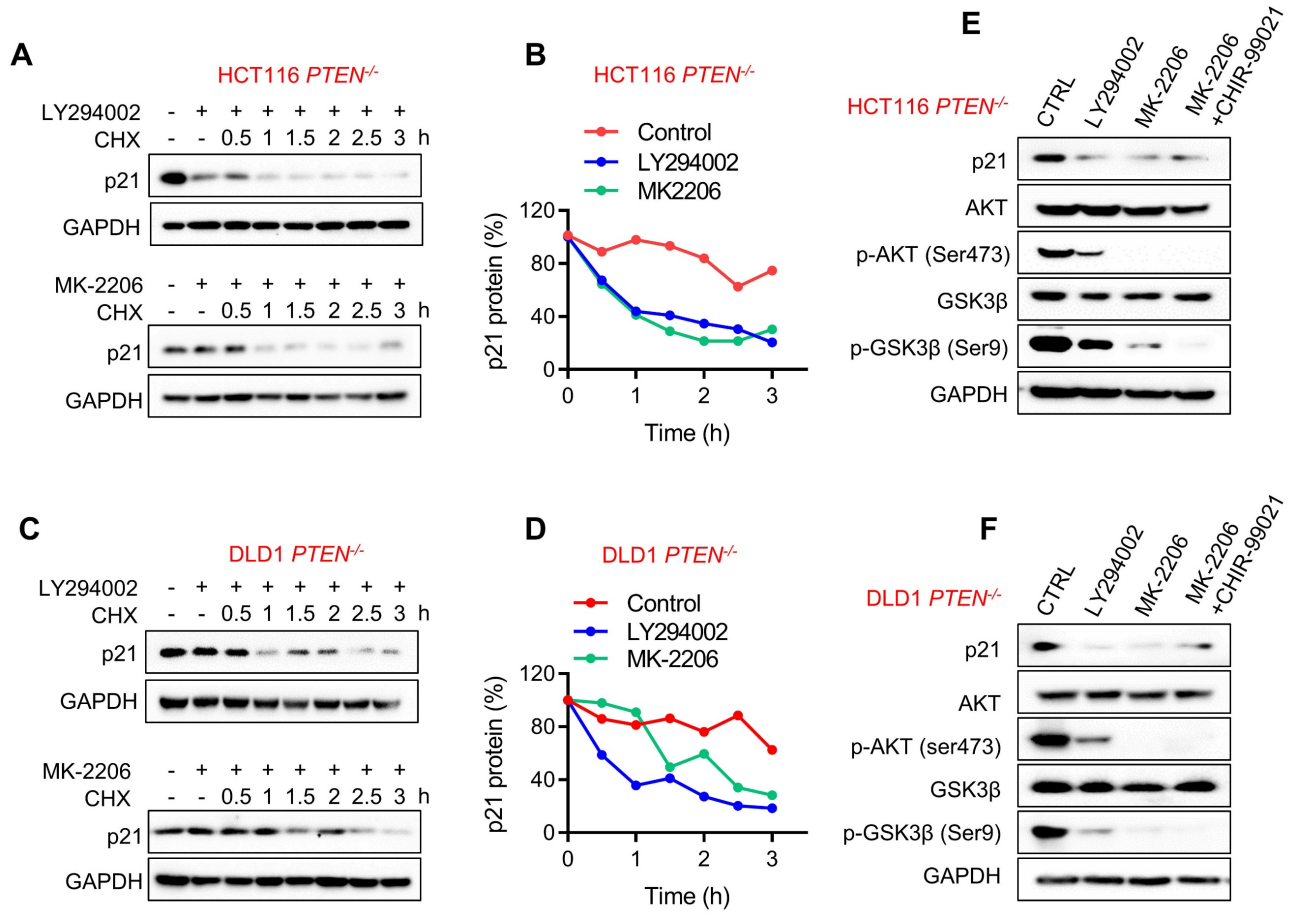
** p21 protein was stabilized through PI3K/AKT activation.** HCT116 (**A**) and DLD1 (**C**) *PTEN^-/-^* cells were incubated with PI3K inhibitor LY-294002 (10 μM) and AKT inhibitor MK-2206 (10 μM) respectively in the presence of cycloheximide (10μM) for the times indicating, and the Western Blot indicated p21protein levels. Graph represented the data from densitometric quantification as ratio of p21/GAPDH and normalized to the "0 CHX control" for each lane; **B** for HCT116, **D** for DLD1. **E, F** Immunoblotting analyzed p21 expression with LY-294002 (10 μM), MK-2206 (10 μM), MK-2206 (10 μM) and GSK3 inhibitor CHIR99021 (10 μM) cotreatment in *PTEN^ -/-^* HCT116 (**E**) and *PTEN^ -/-^* DLD1 (**F**) cells. p-AKT (ser473) and p-GSK3β(s9) were detected for inhibition efficiency confirmation.

It has been reported that AKT could phosphorylate p21 at Ser146 and Thr145 sites, resulting in p21 stabilization and cytoplasmic localization 16, 26. In PTEN absent context, AKT is highly activated, and this could explain the increase in p21 protein stability in PTEN-deficient CRC cells. To verify the AKT-mediated stabilization of p21 protein in PTEN-deficient CRC cells, we used the PI3K inhibitor LY294002 and the AKT inhibitor MK-2206 and evaluated p21 protein half-life in PTEN-deficient CRC cells. Both PI3K and AKT inhibitors significantly shortened the half-life of p21 protein to around 1 h (Fig. 5A-D). These results imply that AKT activation is required for p21 protein stabilization in PTEN-deficient CRC cells. In addition to the direct phosphorylation and regulation of p21 stability, AKT can also indirectly regulate p21 stability by phosphorylating and inhibiting GSK-3β which phosphorylates and promotes p21 protein degradation 27. To rule out this possibility, we tested the effect of AKT inhibitor and GSK-3β inhibitor (CHIR-9902) co-treatment on the p21 level in PTEN-deficient CRC cells. Either PI3K or AKT inhibitor reduced the level of p21 along with the reduced AKT phosphorylation at Ser473 and GSK-3β phosphorylation at Ser9 (Fig. 5E, F), indicating the inhibition of AKT and the activation of GSK-3β. However, GSK-3β inhibitor could not restore p21 protein level when co-treated with the AKT inhibitor, suggesting that GSK-3β is not involved in the regulation of AKT-mediated p21 stabilization.

### BET inhibition suppresses AKT activation, reduces p21 phosphorylation at Thr145 and promotes p21 nuclear import

**Figure 6:**
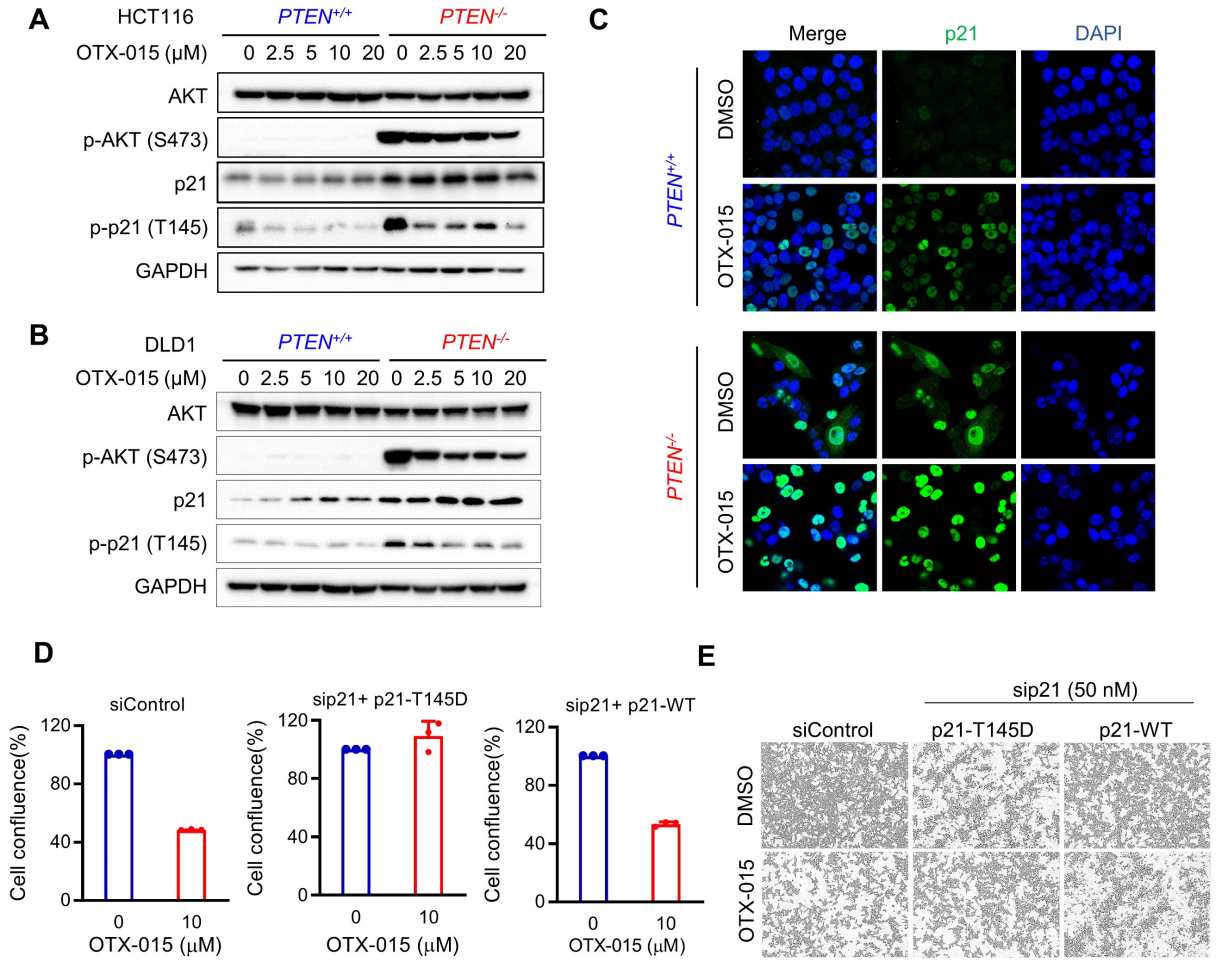
** BETi reduced p21 phosphorylation at Thr145 and promoted p21 nucleus importing by repressing AKT in PTEN-deficient cells. A, B** Western blot illustrating AKT, p-AKT (ser473), p21, p-p21(thr145) levels in HCT116 (**A**) and DLD1 (**B**) PTEN isogenic cell pairs via treatment with OTX-015 for 24 h. **C** Immunofluorescence analysis of p21 localization inHCT116 *PTEN* isogenic cells treated with or without BETi. **D** Endogenous p21 in HCT116 PTEN-deficient cells were repressed by siRNA and the cells were transfected with Thr145 mutant p21 (p21-T145D) and wild type p21 (p21-WT) respectively. The transfected cells were treated with OTX-015 or DMSO for 24 h. The percentages of cell confluence were shown. **E** Cell images represented the cell confluence taken by Incucyte Zoom.

Although p21 is commonly recognized to be a tumor suppressor with its role as a CDK inhibitor, accumulating evidence suggests that p21 possesses duality in cancer regulation depending on its subcellular localization. Nuclear p21 is known to act as a tumor suppressor and cytoplasmic p21 is known to act as an oncogene. Nuclear cytoplasmic shuttling of p21 is regulated by the phosphorylation at Thr145. AKT is one of main kinases that phosphorylate p21 at Thr145 and is known to induce cytoplasmic distribution of p21 16. To explore the role of p21 in the synthetic lethality of PTEN and BET, we analyzed the phosphorylation status and subcellular localization of p21. As expected, high levels of phosphorylated AKT at Ser473 and phosphorylated p21 at Thr145 were observed in PTEN-deficient CRC cells compared to the PTEN-wildtype ones (Fig. 6A, B). BETi dose-dependently reduced AKT phosphorylation and p21 phosphorylation at Thr145 in PTEN-deficient CRC cells, while the total level of p21 was increased by BETi (Fig. 6A, B). In p21 localization study, we observed that overall p21 level was elevated in PTEN-deficient CRC cells in both cytosol and nucleus (Fig. 6C). BETi treatment strongly increased p21 level, preferentially in nuclei, in PTEN-deficient CRC cells (Fig. 6C). These data suggest that, in PTEN-deficient cells, activated AKT promotes p21 phosphorylation at Thr145, leading to the oncogenic conversion of p21 in PTEN-deficient CRC. Inhibition of BET counteracted to this oncogenic conversion of p21 by suppressing AKT and relocating p21 to the nucleus. To further prove this hypothesis, we silenced p21 and re-expressed either wildtype or T145D mutant (phospho-mimetic) version of p21 in PTEN-deficient CRC cells (Supplementary Fig. S3), and analyzed the synthetic lethal effect of BETi. PTEN-deficient CRC cells with wildtype p21 re-expression remained sensitive to BETi, while p21 (T145D) re-expressing cells were strongly resistant to BETi (Fig. 6D, E). These results suggest that p21 phosphorylation at Thr145 by AKT and nuclear-cytoplasmic shuttling of p21 play key roles in BETi-induced synthetic lethality in PTEN-deficient CRC cells.

### BET inhibitor selectively inhibits PTEN*-*deficient tumor growth in mouse xenograft model and patient-derived organoids

**Figure 7:**
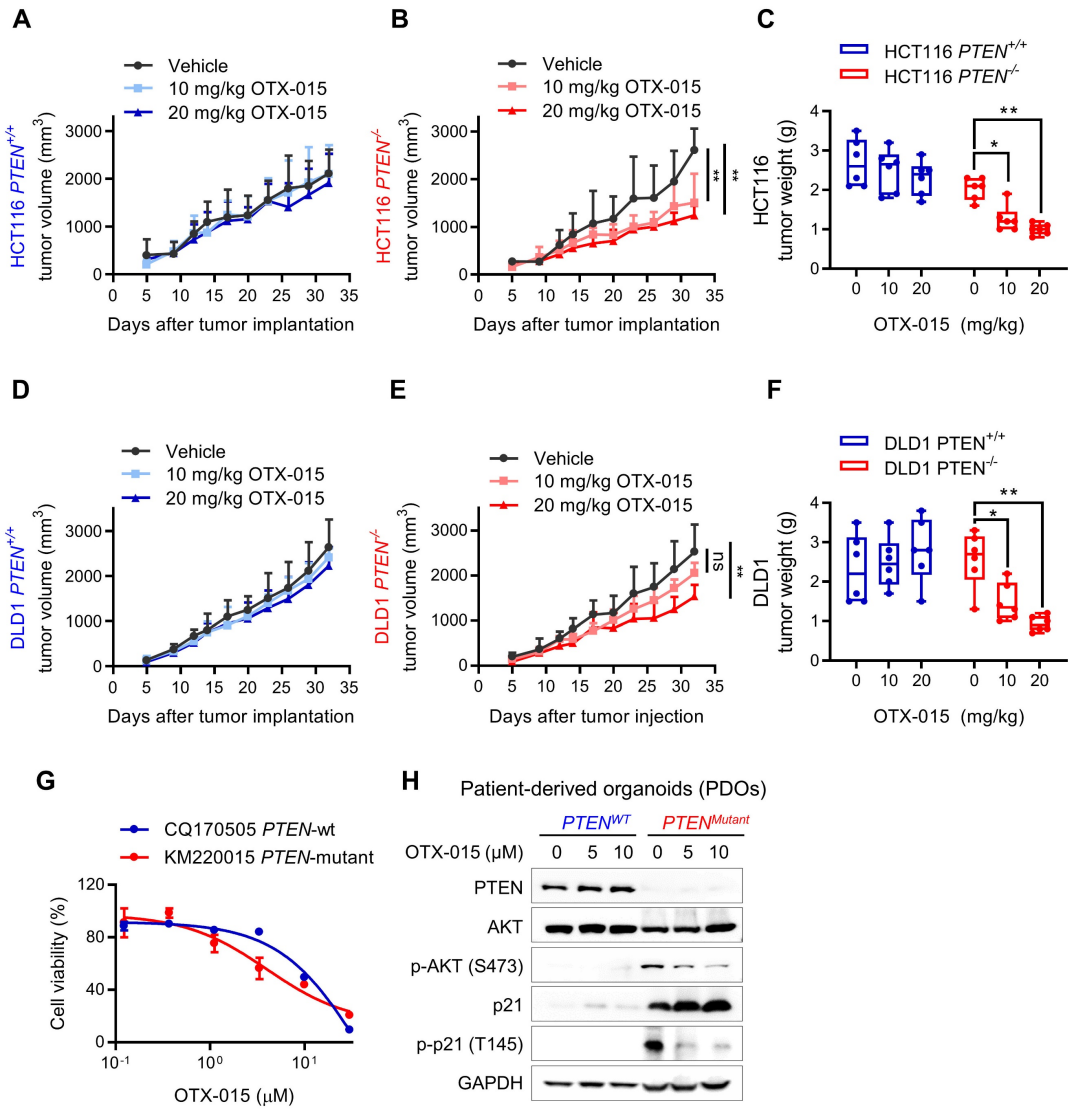
**Verification of PTEN/BET synthetic lethality in vitro.** Tumor growth curves in nude mice bearing HCT116 *PTEN^+/+^* (**A**) or* PTEN^ -/-^* (**B**) xenografts after injection of vehicle or 10 and 20 mg/kg OTX-015. **C** Tumor wet weight analysis of HCT116 *PTEN^+/+^* or *PTEN^ -/-^* tumors treated with vehicle and two doses of OTX-015. **D, E** Tumor growth curves in nude mice bearing DLD1* PTEN^+/+^* (**D**) or *PTEN^ -/-^* (**E**) xenografts after injection of vehicle or the indicated concertation of OTX-015. **F** Tumor wet weight analysis of DLD1 *PTEN^+/+^* or *PTEN^ -/-^* tumors treated with vehicle and two doses of OTX-015. Error bars represent s.d. *P < 0.05, **P < 0.01 between vehicle and BETi treatment groups (n = 6), two-way ANOVA. **G** Viability of patient-derived colorectal tumor organoids following 48 h exposure to the indicated concentrations of OTX-015. **H** Immunoblot showing PTEN, AKT, p-AKT, p21 and p-p21 protein levels in PDOs. GAPDH, loading control.

To assess the therapeutic potential of BETi, we next evaluated the synthetic lethal effects in xenograft mouse model, bearing PTEN-isogenic HCT116 and DLD1 tumors. Mice were given vehicle and two doses (10 and 20 mg/kg) of OTX-015 for a month and the tumor volume was periodically measured. The results showed that PTEN-deficient HCT116 tumors were highly sensitive to BETi treatment, while negligible antitumor effects were observed in PTEN-wildtype HCT116 tumors treated with BETi (Fig. 7A-C). Very similar results were observed in DLD1 tumor xenograft experiments (Fig. 7D-F), demonstrating that BET inhibition is synthetic lethal with PTEN inactivation in vivo. Both doses of OTX-015 did not show any signs of toxicity or body weight change of test animals (Supplementary Fig. S4, B). In addition, we also tested a pair of patient-derived organoids (PDO) from human CRC patients with PTEN-wildtype (CQ170505) and mutant (KM220015). PTEN-mutant PDO culture was more sensitive to BETi than PTEN-wildtype PDO (Fig. 7G). Consistent with the CRC cell line data, BETi treatment reduced phospho-AKT level, increased total p21 protein and reduced the level of phosphorylated p21 at Thr145 in the PDO model (Fig. 7H). These data suggest the potential of PTEN-BET synthetic lethality in the application for PTEN-deficient CRC treatment.

## Discussion

**Figure 8:**
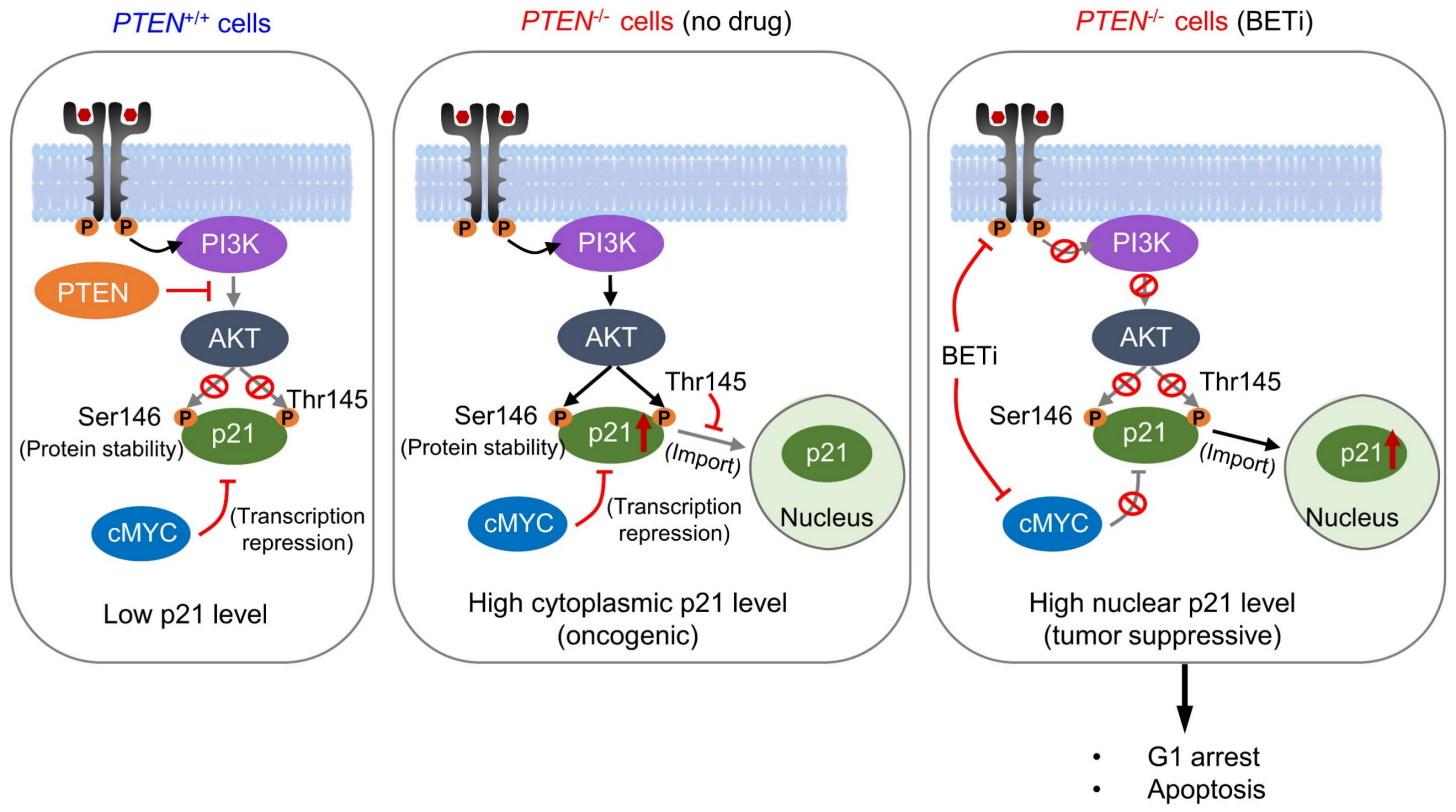
**Schematic illustration delineating mechanism of PTEN and BET synthetic lethality interaction.** AKT was activated in PTEN-deficient cells, thereby stabilized the p21 by phosphorylating, leading to elevated cytosolic p21 level. BETi suppressed AKT signal, dephosphorylating p21 at Thr145, and promoting nuclear translocation of p21. Meanwhile, BETi increased the total level of nuclear p21 by inhibition of MYC. Nuclear p21 induced PTEN-deficient cell G1 cycle arrest and apoptosis.

PTEN is the second most frequently altered tumor suppressor in cancer, next to p53. *PTEN* mutation occurs in 18-30% sporadic CRC 28-30, and its inactivation is associated with the disease advancement of CRC and poor treatment outcome with chemotherapeutics 5, 31. Therefore, exploiting PTEN inactivation has been an important strategy to treat CRC. In order to therapeutically target PTEN inactivation, we conducted a synthetic lethal target screen in CRC with PTEN loss. We found that BET is a synthetic lethal partner of PTEN, which was validated in CRC cell lines and in vivo mouse tumor models. Mechanistic study suggested that PTEN loss promotes p21 stabilization and cytoplasmic retention through AKT activation. High level of cytoplasmic p21 contributes to the oncogenic role of PI3K/AKT in PTEN-deficient CRC cells. BET inhibition has a dual impact on p21, increasing the total level of p21 by inhibiting MYC repression and promoting nuclear import of p21 by inhibiting the upstream pathway of PI3K/AKT. High level of p21 accumulation in the nuclei by BETi induces selective G1 arrest and apoptosis in PTEN-deficient cells (Summarized in Fig. 8).

BET family of proteins consist of two conserved N-terminal bromodomains (BD1 and 2) for epigenetic reading function and an extra terminal domain for the recruitment of transcriptional factors 13. Bromodomain is a protein-protein interaction module that recognizes acetylated lysine residues in histones or transcription factors, thus serving as an epigenetic reader of acetylation signals in gene transcription. BET proteins promote transcription of genes with broad specificity and their dysfunction is involved in many pathological processes, including cancer, immune diseases and CNS disorders 32. Among the BET proteins, BRD2 and BRD3 are known to be involved in cell growth, neurogenesis and cell identify determination, while BRD-T functions in spermatogenesis 33. BRD4 is required for cell proliferation and mitosis, and thus its dysregulation is frequently involved in cancer 34. BRD4 is known to activate the transcription of MYC oncogene and various receptor tyrosine kinases (RTK), contributing to cancer cell proliferation and survival signaling. In our study, we observed that BET inhibition exhibited two independent downstream effects in CRC cells, including MYC repression and AKT inhibition. AKT inhibition by BETi is consistent with previous reports, showing that BETi repressed RTK expression and subsequently inactivated PI3K/AKT pathway 35, 36. Since RTKs serve as an upstream activator of PI3K/AKT, inhibition of AKT phosphorylation by BETi is likely to be attributed to the blockade of RTK expression, such as EGFR family and KIT. We further showed that the two downstream effects of BETi - MYC repression and AKT inhibition - simultaneously target p21 for the protein expression and nuclear translocation, facilitating its tumor suppressive actions.

p21^CIP1/WAF1^ (CDKN1A) is a member of cyclin-dependent kinase inhibitor (CKI), which plays an important role in regulating the cell cycle progression at G1 and S phase. p21 also interacts with proliferating cell nuclear antigen (PCNA) to control S phase DNA synthesis and DNA damage repair 37. Therefore, p21 has been generally recognized as an important tumor suppressor. On the other hand, several studies suggested that p21 has an antagonistic duality feature in controlling cell cycle progression and cell death 10, 38. p21 can promote cell survival through inhibition of multiple cytoplasmic caspases and apoptosis effector molecules 39, 40. It can also promote cell proliferation through facilitating cyclin D/CDK4-6 complex 41. These oncogenic potentials of p21 are attributed to its cytoplasmic localization. In gastric cancer, cytoplasmic p21 expression was significantly correlated with lymph node metastasis, distant metastasis, advanced TNM stage, depth of invasion and overall survival 42.

Cytoplasmic p21 expression also negatively influenced treatment outcome and was correlated with distant metastatic potential of thymic epithelial tumors 43. Furthermore, cytoplasmic p21 was associated with decreased overall survival and relapse-free survival in breast cancer 44. These results suggest that cytoplasmic p21 can promote tumor development and serves as an oncogene 45. The molecular switch to determine nuclear-cytoplasmic shuttling of p21 is the phosphorylation at Thr145 in the C-terminal nuclear localization signal (NLS). Phosphorylation of p21 at Thr145 in the NLS leads to the inhibition of nuclear import, resulting in cytoplasmic retention of p21 16. AKT is a key kinase that phosphorylates p21 at Thr145 and Ser146. AKT phosphorylation of p21 at Thr145 is known to induce cytoplasmic localization and inhibition of binding with PCNA, while the phosphorylation at Ser146 leads to the stabilization of p21 and cell survival. Hence, the phosphorylated, cytoplasmic p21 contributes to the pro-survival actions of PI3K/AKT signal. Consistent with this, our study showed that PTEN-deficient cells have a strong AKT signal, thereby increasing the p21 stability with a high level of phosphorylation at Thr145. Inhibition of BET suppressed AKT signal, reducing p21 phosphorylation at Thr145, causing nuclear translocation of p21. At the same time, BETi-induced inhibition of MYC increased the total level of nuclear p21, promoting tumor suppressive action of p21 in PTEN-deficient CRC cells. Our rescue study further demonstrated the essential role of p21 phosphorylation at Thr145 in synthetic lethality between PTEN and BET.

In conclusion, our study identifies the synthetic lethal interaction between PTEN and BET, and provides the molecular basis of the synthetic lethality where p21 plays a key role in mediating the effect. Further preclinical and clinical investigations of BET inhibitors on personalized therapy for CRC with PTEN molecular subtypes are warranted.

## Supplementary Material

Supplementary figures and tables.
