# Supplementary material for: BET inhibition induces synthetic lethality in PTEN deficient colorectal cancers via dual action on p21CIP1/WAF1
Source: Int J Biol Sci. 2024 Mar 11;20(6):1978–91. doi: 10.7150/ijbs.91867 (PMC11008266; doi:10.7150/ijbs.91867)
Supplement: Supplementary file 1 — Supplementary figures and tables. [file ijbsv20p1978s1.pdf]

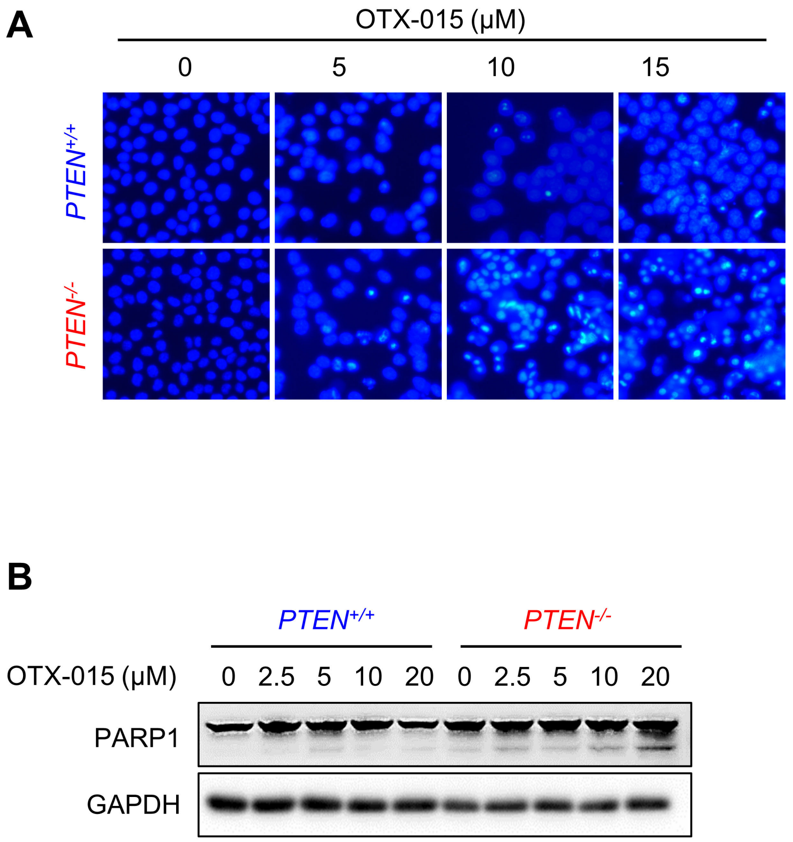

Figure S1 BETi selectively induced apoptotoc in PTEN-deficient cells. A. HCT116 *PTEN* isogenic cell pair were treated with 5, 10,15 μM OTX-015 for 72 h and the nuclei were stained with Hoechst33342. B. Active (cleaved) PARP was used as a marker of apoptosis induction.

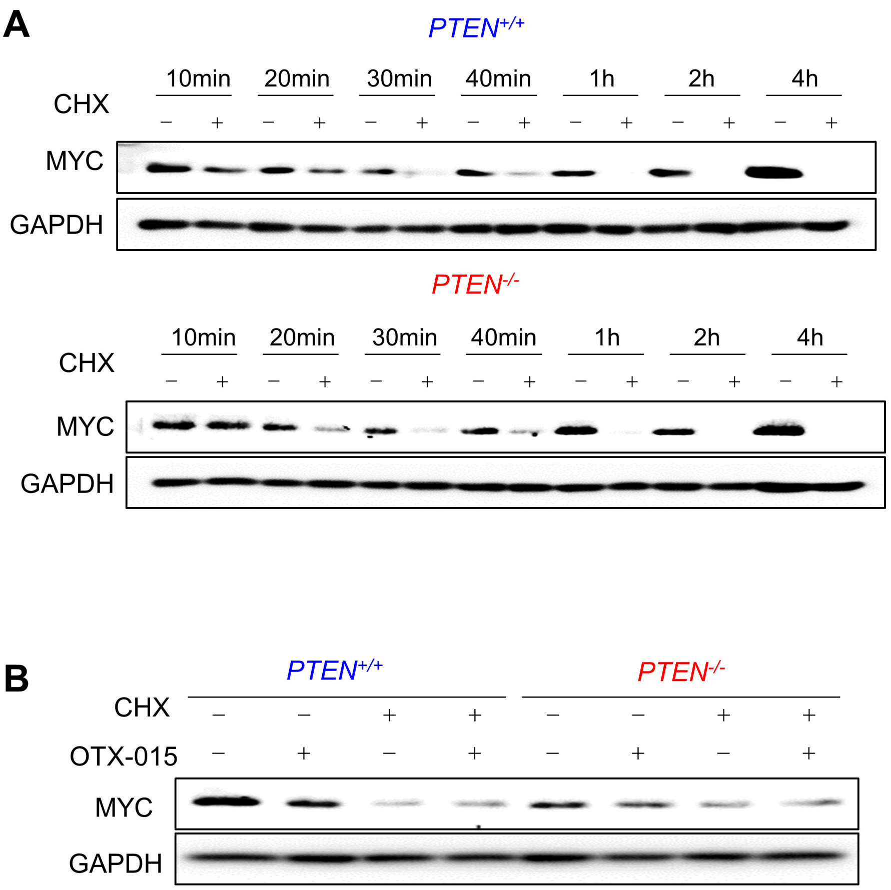

Figure S2 PTEN and BETi did not affect MYC protein stability. Measurement of MYC protein half-life in HCT116 *PTEN* isogenic cells. Cells were treated with or without 10 μM cycloheximide (CHX) and analyzed MYC protein expression for the indicated time points. B. Measurement of MYC protein in *PTEN* isogenic cells with or without OTX-015 (10 μM) treatment.

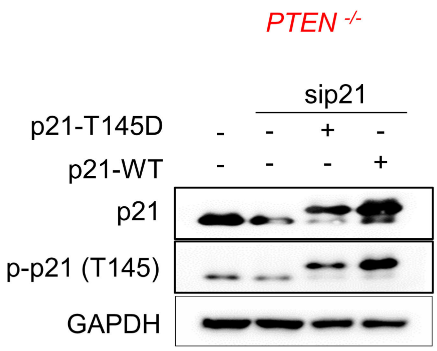

Figure S3 Verification for p21 siRNA, p21-T145D and p21-WT plasmids transfection efficiency. Immunoblot showing p21 siRNA, p21-145A plasmid and p21-wt plasmid transfection efficiency.

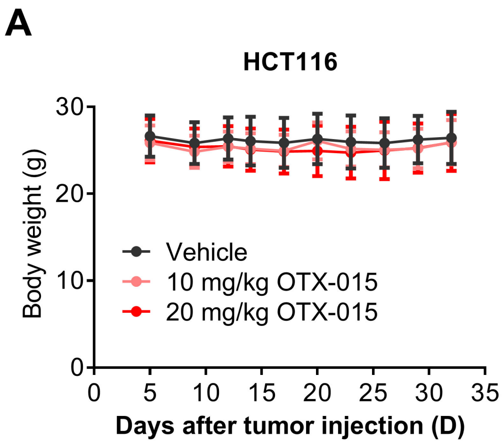

Figure S4 BETi treatment was not toxic to mice. A, B. Mice body weight curves in nude mice treated with vehicle, 10 mg/kg and 20 mg/kg of OTX-015.

Supplementary Table 1. Sequences of siRNAs used in this study

No	Target gene	Suppliers	Sequence
1	siMYC	Integrated DNA Technologies	5’-AUCAUUGAGCCAAAUCUUAAAAAAA-3'
2	sip21	Integrated DNA Technologies	5'-GCCUCUGGCAUUAGAAUUAUUUAAA-3'

Supplementary Table 2. Antibodies use in this study.

No	Primary Antibodies	Suppliers	Catalog No.	Molecular weight (kDa)
1	PTEN (A2B1)	Santa Cruz Biotechnology	sc-7974	55
2	AKT (B1)	Santa Cruz Biotechnology	sc-5298	62
3	p-AKT (ser473)	Cell Signaling Technology	9271	60
4	GAPDH	Santa Cruz Biotechnology	sc-365062	37
5	PARP-1 (H-250)	Santa Cruz	Sc-7150	116
6	cMyc (9E10)	Santa Cruz	Sc-40	65
7	p21 Waf1/Cip1 (12D1)	Cell Signaling Technology	2947	21
8	Phospho-p21 (Thr145)	Thermo fisher	PA5-36677	21
9	GSK-3β (3D10)	Cell Signaling Technology	9832	46
10	Phospho-GSK-3β (Ser9) (5B3)	Cell Signaling Technology	9323	46
11	CDK4 (DCS-35)	Santa Cruz	sc-23896	34
12	Cyclin E	Cell Signaling Technology	sc-377100	53

Supplementary Table 3. Sequences of qPCR primer pairs used in this study.

No	Target gene	Suppliers	Sequence
1	MYC	BGI	Forward 5'-GGCTCCTGGCAAAAGGTCA-3’<br>Reverse 5'-CTGCGTAGTTGTGCTGATGT-3’
2	p21	BGI	Forward 5'-AATACACCAGCAAGCTAGATGC-3’<br>Reverse 5'-AATCAGTTCCGTTCCCCAGAG-3'
3	GAPDH	BGI	Forward 5'-GTGGACCTGACCTGCCGTCT-3’<br>Reverse 5'-GGAGGAGTGGGTGTCGCTGT-3’
